# Christian Science

**Published:** 1900-03

**Authors:** 


Christian Science.—An exposition of Mrs. Eddy's wonderful discovery, including its legal aspects; a plea for children and other helpless sick. By William A. Purrington, Lecturer in the University and Bellevue Hospital Medical College and in the New York College of Dentistry upon Law in Relation to Medical Practice, one of the authors of "A System of Legal Medicine." 194 pages; price, $1.00. New York: E. B. Treat & Co., 241 West 23 Street. 1900.

The frontispiece is a photograph of a child with a gangrened foot treated by a "healer." It is referred to as "an object lesson." On the page facing the preface the following quotation appears: "Christian science demonstrates that the patient who pays whatever he is able to pay for being healed is more apt to recover than he who withholds a slight equivalent for health."—*From preface to Miscellaneous Writings of Mrs. Eddy.*

The book consists of six articles which appeared originally in the *North American Review,* the *Medical Record* and the *New York Sun.* "Four of the papers," says the preface, "deal with the exposition of Mrs. Eddy's teachings, her own account of herself and the status of her cult before the law. Another treats of the educational effect and policy of medical legislation, and the last shows how by enforcement of medical laws not consonant with public opinion the apothecary in England became a general practitioner of medicine."

The subject of Christian Science is treated fairly and seriously. The author admits that there are intelligent, honest people who believe and practice Christian Science. The questions submitted to Mrs. Eddy and her high apostle, Mr. Carol Norton, had not been answered at the time the book went to press. They were asked, for instance, if they could counteract by their arguments the effects of morphine, atropine or strychnine. On page 113, Mr. Norton was asked: "I said to that gentleman, if a lad should accidently sever an artery and surgical aid were accessible, would you presume to set that aid aside, and essay to staunch the gush of arterial blood by your mental processes alone?" "The question," says the author, "presented a dilemma; to say that he would not do so implied lack of faith in the doctrine he teaches and practices for a livelihood; to admit that he would do so, would confess willingness to commit a felony. Is it any wonder that he preferred 'to shelve' the question?"

Any one desiring to familiarize himself with the subject of Christian Science treated fairly and logically would do well to send to the publishers for a copy of the above work. T. J, B.

---

TWENTIETH CENTURY PRACTICE.—The JOURNAL has sixteen volumes of this great work, new, which we will sell for $40—that is, $2.50 per volume; the publishers' price is $5.00 per volume, and sold only on subscription. We don't pay express.

